# SCN5A Cardiomyopathy: from Ion Channel Dysfunction To Clinical Disease

**DOI:** 10.1007/s11886-025-02298-5

**Published:** 2025-10-09

**Authors:** Astrid B.M. Heymans, Lorenzo Bianchi, Paul G.A. Volders, Saskia N. van der Crabben, Job A.J. Verdonschot

**Affiliations:** 1 Department of Cardiology, Cardiovascular Research Institute Maastricht, University of Maastricht & Maastricht University Medical Center, Maastricht, The Netherlands; 2 Department of Internal Medicine, University of Genova, Genova, Italy; 3 Department of Clinical Genetics, Maastricht University Medical Center, P. Debyelaan 25, Maastricht, 6229 HX The Netherlands; 4 European Reference Network for Rare, Low Prevalence and Complex Diseases of the Heart, (ERN GUARD-Heart), Maastricht, The Netherlands

**Keywords:** SCN5A, Cardiomyopathy, Genetics, Sodium channels, Review

## Abstract

**Purpose of Review:**

Although *SCN5A* variants are an established cause of arrhythmia and conduction disease, their association with dilated cardiomyopathy (DCM) is less studied. This review summarizes recent insights into *SCN5A*-related cardiomyopathy, focusing on genotype-phenotype correlations, overlap with arrhythmia, and implications for management.

**Recent Findings:**

Both gain- and loss-of-function *SCN5A* variants are associated with cardiomyopathy, found in 0.5–0.9% of DCM cases. Presentation ranges from isolated DCM to overlap phenotypes, in both pediatric and adult patients. High variability and intrafamilial heterogeneity suggest pleiotropic effects and variable penetrance. High prevalence of arrhythmias and conduction disease suggests the DCM phenotype may be mediated by electrical disturbances. However, functional studies and cases without prior arrhythmia suggest *SCN5A* variants may directly contribute to structural myocardial changes.

**Summary:**

*SCN5A*-related cardiomyopathy is a rare disorder at the intersection of structural and electrical heart disease. Genotype-informed strategies, including arrhythmia management, and early cascade genetic screening are clinically relevant. Further research should address *SCN5A*-specific risk management in DCM patients.

## Introduction

The *SCN5A* gene encodes the pore-forming alpha-subunit of the cardiac sodium channel (NaV1.5), which mediates inward sodium current essential for action potential initiation and propagation and normal electrical conduction. Pathogenic and likely pathogenic (P/LP) *SCN5A* variants impair channel expression and function and underlie a spectrum of disorders, including long QT syndrome type 3 (LQT3), Brugada syndrome type 1 (BrS1), progressive cardiac conduction disorders (PCCD), sick sinus syndrome (SSS), atrial standstill, familial atrial fibrillation, and multifocal ectopic Purkinje-related premature contractions (MEPPC). However, they can also cause cardiomyopathies, most notably dilated cardiomyopathy (DCM).

*SCN5A* variants resulting in gain of function of NaV1.5 increase sodium current and can typically cause LQT3 and MEPPC. Loss-of-function variants reduce sodium influx and underlie BrS), SSS, atrial standstill, and PCCD. Familial atrial fibrillation has been linked to both loss- and gain-of-function variants. Both functional effects have been observed in DCM-associated *SCN5A* variants [1, 2]. A single *SCN5A* variant can also result in a sodium channel overlap syndrome, with multiple phenotypes associated with both gain and loss of sodium channel function occurring within one individual, or within one family [3, 4]. For cardiomyopathies, cases of isolated DCM, as well as overlapping phenotypes such as concurrent LQT3 and DCM, have been identified [5, 6].

This review summarizes current knowledge on the role of *SCN5A* variants in cardiomyopathy, and focuses on implications for clinical management, emerging directions in research, with a focus on recent findings and developments.

### Spectrum of SCN5A-related cardiomyopathies

P/LP variants in *SCN5A* are associated with different types of cardiomyopathies.

The relationship between *SCN5A* variants and DCM is well-established, but it is rare and characterized by marked heterogeneity [7]. It is often accompanied by conduction system disease, frequent premature ventricular complexes (PVCs), atrial and ventricular arrhythmias, and in rare cases, specific variants can show overlap with other cardiac channelopathies such as LQT3 [6].

Beyond DCM, *SCN5A* variants have been reported in patients diagnosed with arrhythmogenic cardiomyopathy (ACM), including right- and left-dominant forms [8–10]. Up to 18 different *SCN5A* variants, particularly those within the transmembrane voltage-sensing domains of NaV1.5, have been linked to ACM [10, 11]. *SCN5A*-related non-dilated left ventricular cardiomyopathy has sporadically been reported. Lastly, a few pediatric cases have noted *SCN5A* variants in patients exhibiting non-compaction, now redefined as hypertrabeculation and considered a morphological trait rather than a distinct cardiomyopathy [7, 12]. Overall, the spectrum of *SCN5A*-related cardiomyopathies underscores the complexity of classifying these entities. Unlike sarcomeric and desmosomal genes, *SCN5A* is not a classical cardiomyopathy gene, and clinical manifestations often overlap with arrhythmia syndromes. This highlights the need for novel gene-oriented classifications.

### Inheritance and clinical characteristics of SCN5A-related cardiomyopathy in adults

*SCN5A* variants are inherited in an autosomal dominant manner and demonstrate incomplete penetrance and variable expressivity, even among members of the same family [2, 13].

**Fig. 1:**
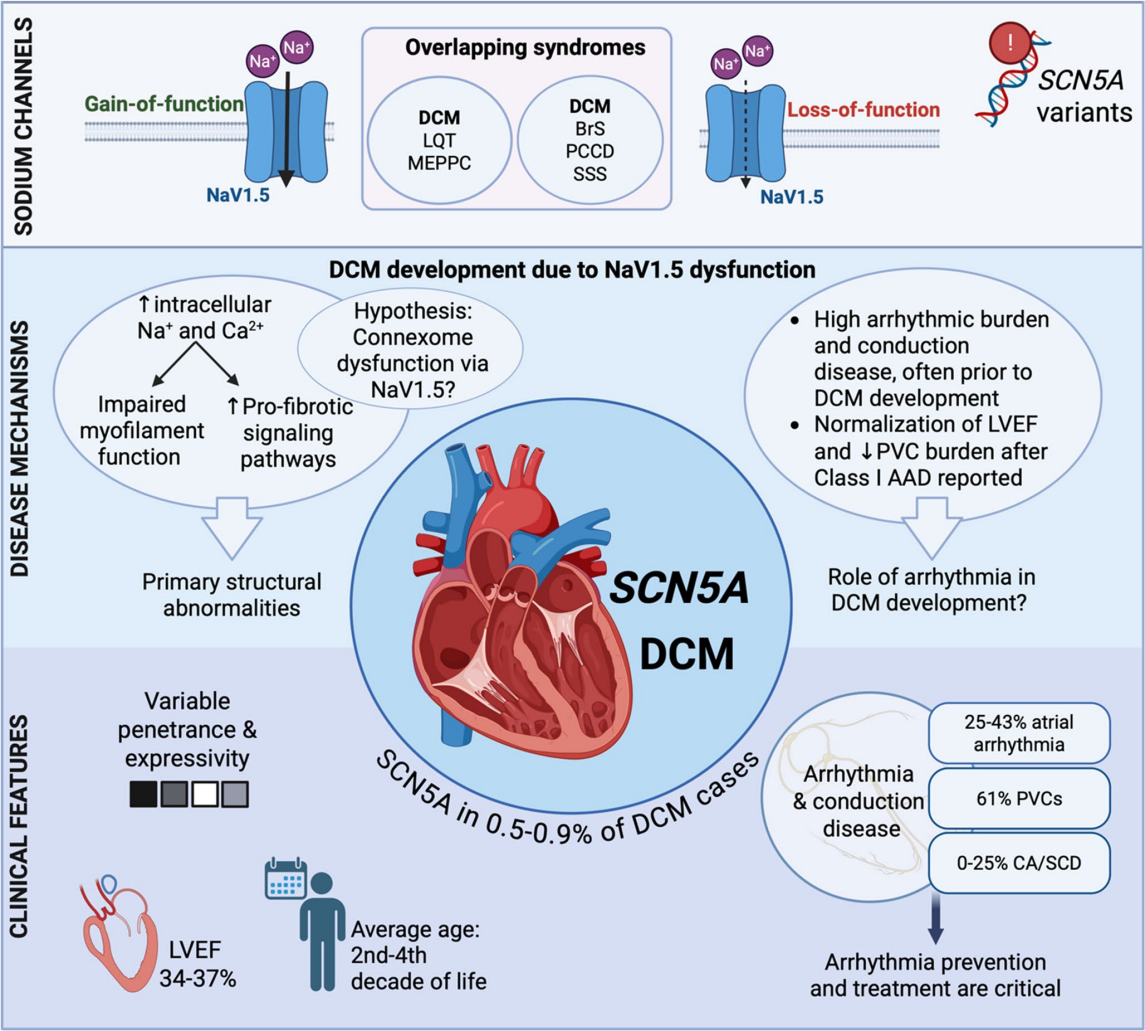
Overview figure of *SCN5A*-related DCM Numbers are based on the SCN5A-DCM probands of Peters et al. and Hermida et al. [5, 10]. (Created in BioRender. Heymans, A. (2025) https://BioRender.com/tw1pqtw.)

The finding of P/LP variants in *SCN5A* in patients with DCM is rare, with a prevalence of 0.5–0.9% in adults in Europe and the United States (compared to e.g., up to 17% for P/LP variants in *TTN*) [14–16]. The age of onset in SCN5A-related DCM is highly variable, ranging from described pediatric cases to cases with onset at the age of 66 years [5, 6, 10]. The mean age of onset is estimated in the second to fourth decades of life (Fig. 1) [10]. To our knowledge, no observational cohorts of *SCN5A*-related cardiomyopathy patients have reliably described left ventricular dysfunction. In two systematic reviews [5, 10], *SCN5A*-DCM probands showed a mean LVEF of 34–37%, with reduced function in 26 of 27 patients, except one with borderline normal function (LVEF 51%). These findings are based on very small numbers and may reflect selection and publication bias. Further studies are needed to clarify the extent of LV dysfunction. *SCN5A* variants have been described to cause early-onset conduction disease followed by later DCM development, with conduction abnormalities often preceding the onset of DCM by approximately 5–10 years [2, 10, 17, 18]. This suggests that DCM may, in some cases, arise secondary to chronic electrical disturbances. However, more research is needed to determine whether conduction disease contributes causally to DCM development, or rather represents as an early manifestation of the cardiomyopathy due to greater sensitivity to genotypic abnormalities.

Clinical presentation is heterogeneous, but arrhythmic abnormalities are the most consistent clinical feature. Patients may present with syncope due to ventricular arrhythmias or conduction disturbances, sinus node dysfunction, atrial standstill, or atrioventricular block. Palpitations frequently reflect an underlying electrical disturbance, arising from atrial or ventricular arrhythmias, such as ventricular ectopic activity. Furthermore, dyspnea and fatigue are common symptoms in patients with left ventricular dysfunction. Notably, sudden cardiac death remains a major risk and can occur as the first manifestation of the disease, even in previously asymptomatic individuals who are genotype-positive [5, 10].

Peters et al. conducted a Genotype-phenotype analysis of 173 patients with a positive phenotype from 29 *SCN5A*-DCM families, encompassing 18 unique variants and 143 confirmed *SCN5A*-heterozygotes [10]. In all the 29 probands, conduction disease or arrhythmias were the initial clinical features, with 66% also having DCM at the time of clinical diagnosis. Among probands without DCM at initial evaluation, they manifested DCM later during follow-up. Probands who did not develop DCM but only exhibited an arrhythmic phenotype always had at least one relative who developed DCM. Among all affected individuals, including probands and affected relatives, 52% exhibited PVCs (percentage of PVC burden was not specified) or ventricular arrhythmias, 33% had atrial arrhythmias, 9% presented with atrioventricular block before the age of 70, and only 2% had prolonged QT intervals. Severe left ventricular dysfunction (left ventricular ejection fraction (LVEF) < 20%), heart transplantation, or death from heart failure occurred in 8% of cases. Additionally, cardiac arrest or sudden cardiac death occurred in 11%, reflecting the substantial arrhythmogenic risk associated with *SCN5A*-related DCM, though this may also be influenced by advanced heart failure irrespective of genotype [10].

Table 1 provides an overview of clinical features in patients with *SCN5A*-related disease, specifically highlighting *SCN5A*-DCM probands described in the systematic reviews by Peters et al. and Hermida et al. [5, 10] All reported cases in Peters et al. with variant c.656G > A (*n* = 6) exhibited a DCM phenotype. In contrast, the variant c.665G > A (*n* = 73) was associated with a high arrhythmic burden, with 70% demonstrating ventricular arrhythmia or PVCs, and 58% developed DCM. The c.3820G > A variant (*n* = 25) appeared to carry a lower risk for DCM, with only 24% of cases showing DCM. These examples illustrate the variant-specific differences in DCM expression among SCN5A-related cases. Peters et al. did not report on BrS, the results of Hermida et al. revealed that none of the DCM probands exhibited a Brugada phenotype.

**Table 1:** Major evidence for *SCN5A*-related disease and DCM genotype-phenotype correlations

Study	Patients	Genetic variant	Protein effect	Channel location	Sex	Symptoms onset	DCM	Age of DCM onset	Rhythms and AVB	PVC/VA	AA	CA/SCD	Other electrical disorders	EF (%) at DCM onset	Relatives with DCM/SCD
Peters et al. 2022 [10], systematic review	**SCN5A-related diseases** ***n* = 173**	***n* = 18**	***n* = 18**	***n* = 18**	**M *n* = 94** **(58%)**	**NA**	**61%**	**NA**	**AVB 10%**	**47% (PVC 49%, VA 25%)**	**33%**	**11%**	**BrS NA/ not described**	**NA**	
	*n* = 6	c.656G>A	p.R219H	D1 S4	NA	NA	6 (100%)	NA	AVB 17%	100%	17%	16%		NA	
	*n* = 73	c.665G>A	p.R222Q	D1 S4	NA	NA	42 (58%)	NA	AVB 1%	70%	29%			NA	
	*n* = 1	c.674G>C	p.R225P	D1 S4	NA	22 w	1 (100%)	NA		100%	100%	33%		Reduced	
	*n* = 6	c.2440C>T	p.R814W	D2 S4	NA	NA	4 (67%)	NA		50%	50%			NA	
	*n* = 1	c.611C>A	p.A204E	D1 S3	F (100%)	18	1 (100%)	NA		100%	100%			NA	
	*n* = 20	c.638G>A	p.G213D	D3 S3	NA	NA	5 (25%)	NA	AVB 10%	80%	40%	25%		30%	
	*n* = 4	c.2482C>T	p.L828F	D2 S4-S5 L	F (100%)	Adoles-33y	3 (75%)	NA		75%		50%		NA	
	*n* = 4	c.4516_4524del	p.Q1506_P1508del	D3-D4 L	NA	NA	2 (50%)	NA	AVB 25%	25%				NA	
	*n* = 25	c.3820G>A	p.D1274N	D3 S3	NA	NA	6 (24%)	29-55y	AVB 16%		52%			25-47%	
	*n* = 1	c.4024A>G	p.I1342V	D3 S5	M (100%)	4 y	1 (100%)	4 y		100%	100%	14%		35%	
	*n* = 7	c.4780G>C	p.D1594H	D4 S3	NA	NA	1 (14%)	NA			14%	17%		Normal	
	*n* = 6	c.589G>C	p.D197H	D1 S3	NA	NA	4 (67%)	NA	AVB 67%	33%	83%			NA	
	*n* = 3	c.1111C>G	p.Q371E	D1 S5-S6 L	NA	NA	3 (100%)	NA						NA	
	*n* = 3	c.2184_2186del	p.L729del	D2 S1	NA	NA	1 (33%)	NA						NA	
	*n* = 4	c.2550-2551insTG	p.F851Cfs*19	D2 S5	NA	NA	2 (50%)	NA	AVB 75%	50%				NA	
	*n* = 4	c.3832G>A	p.V1278I	D3 S3	M (50%)	NA	2 (50%)	NA		25%	25%			NA	
	*n* = 4	c.4557C>G	p.F1519L	D3-D4 L	NA	NA	4 (100%)	NA		25%				Reduced	
	*n* = 1	c.4639G>C	p.E1547Q	D4 S1	M	15 y	1 (100%)	15 y		100%	100%			48%	
	***SCN5A*-DCM** **probands** ***n*** **= 23**	***n* = 18**	***n* = 18**	***n* = 12**	**M *n* = 13** **(65%)**	**Mean 21 y**	**100%**	**Mean 23 y**	**AVB 17%**	**83% (PVC 61%, VA 65%)**	**43%**	**0%**	**BrS NA/ not described**	**Mean 36%**	
		c.656G>A	p.R219H	D1 S4	M	NA	DCM	29 y	AVB	NSVT	AFlu			49%	5 DCM
		c.665G>A	p.R222Q	D1 S4	M	25 y	DCM	NA	IVR	PVC + VT				20%	2 DCM (2 SCD), 1 SCD
		c.665G>A	p.R222Q	D1 S4	F	10 y	DCM	13 y	JB	PVC + NSVT				32%	3 DCM, 3 SCD
		c.665G>A	p.R222Q	D1 S4	F	27 y	DCM	27 y		PVC + NSVT	AF			35%	4 DCM
		c.665G>A	p.R222Q	D1 S4	M	19 y	DCM	19 y		PVC + VT	AF			37%	1 DCM
		c.665G>A	p.R222Q	D1 S4	F	22 y	DCM	22 y			PAC			35%	5 DCM, 1 SCD
		c.665G>A	p.R222Q	D1 S4	M	38 y	DCM	38 y		PVC				NA	3 DCM (1 SCD)
		c.665G>A	p.R222Q	D1 S4	M	41 y	DCM	41 y		PVC	PAC			20%	NA
		c.665G>A	p.R22Q	D1 S4	F	27 y	DCM	27 y		PVC + NSVT	AF			35%	NA
		c.674G>C	p.R225P	D1 S4	NA	22 w	DCM	22 w		PVC	AT			Reduced	NA
		c.2440C>T	p.R814W	D2 S4	F	23 y	DCM	23 y		NVST	AFlu			30%	0
		c.2440C>T	p.R814W	D2 S4	M	36 y	DCM	56 y		PVC + VT	AF-AFlu			35%	1 DCM, 2 SCD
		c.611C>A	p.A204E	D1 S3	F	18 y	DCM	18 y	JB	PVC				30%	0
		c.2482C>T	p.L828F	D2 S4-S5 L	F	24 y	DCM	24 y		PVC + NSVT				23%	1 SCD
		c.4516_4524del	p.Q1506_p.1508del	D3 D4 L	NA	Infant	DCM	infant	AVB					37%	1 DCM, 2 SCD
		c.4024A>G	p.I1342V	D3 S5	M	4 y	DCM	4 y		PVC + NSVT	AF			35%	0
		c.589G>C	p.D197H	D1 S3	NA	Infant	DCM	infant	AVB + SND	VT			LQTS	Reduced	1 DCM (1 SCD), 1 SCD
		c.1111C>G	p.Q371E	D1 S5-S6 L	M	12 y	DCM	> 12 y						NA	2 DCM (1 SCD)
		c.2184_2186del	p.L729del	D2 S1	M	NA	DCM	49 y						NA	2 SCD
		c.2550-2551insTG	p.F851Cfs*19	D2 S5	M	NA	DCM	32 y	AVB	VT			LQTS	35%	1 DCM
		c.3832G>A	p.V1278I	D3 S3	M	NA	DCM	50 y		NSVT	AF			51%	1 DCM
		c.4557C>G	p.F1519L	D3-D4 L	M	NA	DCM	25 y		PVC + NSVT	PAC			35%	3 DCM
		c.4639G>C	p.E1547Q	D4 S1	M	NA	DCM	15 y		PVC + NSVT	AFlu			48%	NA
Hermida et al. 2024 [5], systematic review^+^	**SCN5A-related diseases *n* = 24**	***n* = 8**	***n* = 8**	***n* = 8**	**NA**	**NA**	**49%**	**NA**	**PCCD 35%**	**PVC 25%, VA NA**	**12%**	**13%**		**NA**	
	*n* = 1	c.1153G>A	p.A385T	DI S5-S6	F	22 y	1 (100%)	22 y		PVC 100%		100%		30%	
	*n* = 2	c.2182G>A	p.V728I	D2 S1	NA	NA	1 (50%)	NA	PCCD 50%				BrS 50%	NA	
	*n* = 3	c.2435T>C	p.L812P	D2 S4	NA	NA	2 (66%)	NA	SSS 33%	MEPPC 100%				NA	
	*n* = 1	c.250G>A	p.D84N	Cyt N Term	F	52 y	1 (100%)	52 y	PCCD 100%					30%	
	*n* = 3	c.4472A>G	p.Q1491R	D3-D4 L	NA	NA	1 (33%)	NA	SSS 100%		AF 33%			NA	
	*n* = 1	c.4859C>T	p.T1620M	D4 S3-S4	M	51 y	1 (100%)	51 y	PCCD 100%					45%	
	n = 2	c.5085G>C	p.Q1695H	D4 S5-S6	NA	NA	1 (50%)	NA				50%	BrS 50%	NA	
	n = 3	c.673C>T	p.R225W	D1 S4	NA	NA	1 (33%)	NA	PCCD 33%		33%		BrS 66%	NA	
	***SCN5A*-DCM** **probands** ***n* = 8**	***n* = 8**	***n* = 8**	***n* = 8**	**M *n* = 3** **(43%)**	**Mean 40 y**	**100%**	**Mean 40 y**	**PCCD 50%**	**NA**	**25%**	**25%**		**Mean EF 34%**	
		c.1153G>A	p.A385T	DI S5-S6	F	22 y	DCM	22 y		PVC		CA/SCD		30%	0
		c.2182G>A	p.V728I	D2 S1	F	56 y	DCM	56 y	PCCD					40%	0
		c.2435T>C	p.L812P	D2 S4	NA	NA	DCM	NA	SSS	MEPPC				NA	1 DCM
		c.250G>A	p.D84N	Cyt N Term	F	52 y	DCM	52 y	PCCD					30%	0
		c.4472A>G	p.Q1491R	D3-D4 L	F	19 y	DCM	19 y	SSS		AF			40%	0
		c.4859C>T	p.T1620M	D4 S3-S4	M	51 y	DCM	51 y	PCCD					45%	0
		c.5085G>C	p.Q1695H	D4 S5-S6	M	16 y	DCM	16 y				CA/SCD		30%	0
		c.673C>T	p.R225W	D1 S4	M	66 y	DCM	66 y	PCCD		AA			20%	0

All means were reported by the review, or calculated based on the available data, excluding missing values. When data was insufficient, no mean was calculated

+ Patients reported by Peters et al. have been excluded in the summary of Hermida et al. to avoid duplicate reporting and redundancy

Abbreviations: *AA* atrial arrhythmias; *Adolesc* adolescent; AF = atrial fibrillation; *AFlu* atrial flutter; *AT* atrial tachycardia; *AVB* atrioventricular block; *CA* cardiac arrest death; *CMP* cardiomyopathy; *DCM* dilated cardiomyopathy; *EF* ejection fraction; *JB* junctional bradycardia; *L* linker; *LQTS* long QT syndrome; *MEPPC* multifocal ectopic premature Purkinje-related contractions; *M* male; *F* female; *D* domain; *NA* not available; *NSVT* not ventricular sustained tachycardia; *P +* phenotype positive; *PAC* premature atrial complexes; *pts* patients; *PCCD* progressive cardiac conduction disease; *PVC* premature ventricular complexes; *S* segment; *SCD* sudden cardiac death; *SND* sinus node dysfunction; *SSS* sick sinus syndrome; *VA* ventricular arrhythmias; *VT* ventricular tachycardia; *w* weeks; *y* years; *3*^*rd*^ 3^rd^ degree

### Variable penetrance of SCN5A: familial insights

Certain *SCN5A* variants have demonstrated high penetrance: the A1180V loss-of-function variant was reported in a Chinese family with high penetrance. It had an age-dependent penetrance, where an atrioventricular block typically preceded DCM development [2]. Similarly, four gain-of-function *SCN5A* variants (L812P, R814W, T1779M, R222Q) linked to multifocal Purkinje-related extrasystoles and often also DCM have been reported, which exhibited high familial penetrance, affecting seven out of eight relatives who carried the variant [5]. Conversely, the L889V loss-of-function variant displayed variable penetrance and expressivity. This variant primarily manifested as conduction system disorders and, in some cases, was associated with BrS, DCM, or ventricular arrhythmias. It exhibited high penetrance in two families with variable phenotypic expression. In a third family, penetrance was lower, affecting only two individuals among the genotype-positive relatives with different phenotypes, of which one manifested an overlap phenotype including DCM [13]. The G1406R loss-of-function variant, reported in a large French family with high penetrance and pleiotropic expression, manifested as either BrS or cardiac conduction defects [19]. Penetrance of *SCN5A* variants is often age-related and may be influenced by other factors. It is suggested that variable and region-specific expression of the mutant allele, along with genetic modifiers, environmental factors, and sex differences may influence the observed phenotypic variability [19]. The illustrated incomplete and age-dependent penetrance suggests the role of potential “second hit” mechanisms or protective factors [20]. Accordingly, cascade genetic screening is crucial for the identification of at-risk relatives.

### Clinical characteristics of pediatric SCN5A-related cardiomyopathy

Pediatric patients with P/LP SCN5A variants exhibit a broad phenotypic spectrum, ranging from asymptomatic carriers to severe disease. In a cohort of 442 children (≤ 16 years) with confirmed P/LP SCN5A variants, 67.9% underwent genetic testing as part of family screening and were asymptomatic [6]. Among the phenotype-positive patients, a DCM phenotype was rare. At baseline, only three patients had isolated DCM, all asymptomatic at the time of diagnosis with a mean age of seven years. An additional four cases showed DCM as part of an overlap phenotype (LQT3 and DCM: n = 1, PCCD and DCM: n = 3). In another cohort of 41 genotyped pediatric DCM cases, only one patient (2.4%) had a P/LP SCN5A variant (R222Q) [21]. This patient was identified via genetic testing after identification of a paternal P/LP SCN5A variant. He developed severe, but asymptomatic DCM by age 17 (LVEF 12.5%) after PVCs were detected at age 10. This variant is associated with MEPPC, causing hyperexcitability of Purkinje fibers and associated with cardiac dilatation [22]. Furthermore, in a cohort of 25 children with left ventricular non-compaction cardiomyopathy, two (8.0%) had an SCN5A variant, diagnosed at the age of 10.3 and 12.6 years; one required heart transplantation [12]. It is not provided whether this variant was de novo or inherited from an affected parent.

### Insights into SCN5A-related cardiomyopathy through comparison with LMNA

Both *LMNA* and *SCN5A* variants are associated with a broad phenotypic DCM spectrum, often accompanied by conduction defects, atrial fibrillation, and ventricular arrhythmias. In both entities, electrical abnormalities often seem to precede the onset of DCM. In *LMNA*, conduction disturbances and arrhythmias typically occur a median of seven years before DCM onset [23], mirroring findings in *SCN5A*-related DCM [2, 10].

*LMNA* encodes lamins A and C, key nuclear membrane proteins involved in supporting nuclear integrity and regulating chromatin organization and gene expression [24, 25]. P/LP *LMNA* variants are a well-established cause of DCM, typically inherited in an autosomal dominant manner with high cardiac penetrance (~ 84% of carriers) [26, 27]. The mean age at diagnosis is 37.6 ± 13.2 years, with an average left ventricular ejection fraction of 33.9% ± 10.3 [26] Similarly, *SCN5A*-related DCM is estimated to manifest between the second and fourth decades of Life, with mean LVEF reported between 34 and 37% [2, 10, 11, 28].

*LMNA* variants are commonly associated with extensive myocardial fibrosis, with late gadolinium enhancement (LGE) detected in ~ 50% of *LMNA*-DCM cases on cardiac magnetic resonance (CMR) imaging, with a global LGE extent of 3.23% in *LMNA* carriers with a reduced ejection fraction [29, 30]. In contrast, LGE was observed in only 1 of 11 (9.1%) *SCN5A*-related cases [10], suggesting a lower fibrotic burden, though the available data is scarce.

Both Gene groups carry significant arrhythmic risk. Ventricular arrhythmias were reported in 37.5% of *LMNA* and 26% of *SCN5A* DCM cases, with sudden cardiac death occurring in 10.7% of the latter [10, 26]. Notably, *SCN5A* DCM with frequent multifocal PVCs responds well to antiarrhythmic therapy: in one series, 87% (20 of 23) of patients treated with sodium channel blockers showed complete resolution of PVCs and normalization of LVEF [10]. This suggests that *SCN5A*-related DCM may, in some cases, be secondary to electrical dysfunction rather than solely a primary structural cardiomyopathy, although no studies have clearly described the response of *SCN5A* DCM to heart failure therapy. In contrast, *LMNA*-related DCM development has not been associated with high PVC burden and no reversibility of dysfunction with AAD has been described, likely due to its extensive fibrosis. *LMNA*-related DCM however demonstrated limited left ventricular reverse remodeling in response to heart failure therapy [31].

In summary, while *SCN5A* and *LMNA*-related cardiomyopathies have some similar phenotypical features, such as early conduction disease, high arrhythmic burden, and similar age of onset, important differences exist. *LMNA* variants are associated with a highly penetrant and well-characterized phenotype, whereas *SCN5A*-related disease shows more variable penetrance. *SCN5A*-related DCM appears to have less fibrosis, and cases have been described that show great reversibility of left ventricular function after AAD, suggesting a potentially more dynamic phenotype. These differences have clinical implications. *LMNA* variants often warrant early, more aggressive preventive measures such as preventative implantable cardioverter defibrillator (ICD) placement, whereas *SCN5A*-related DCM may benefit from close arrhythmia surveillance, early rhythm control, and assessment of reversibility. However, genotype-phenotype associations are well-investigated and described for *LMNA*, while it is less well defined for *SCN5A. SCN5A* needs to be studied further to determine the most effective management strategies.

### Overlap between SCN5A-related Cardiomyopathy and Arrhythmic/conduction Disorder

A single variant can manifest as different phenotypes across several relatives and within a single individual. In a family with a history of sudden cardiac death, carriers of the same gain-of-function *SCN5A* variant (V1323L) exhibited distinct phenotypes: one was diagnosed postmortem with ARVC, while another presented with LQT and mild myocardial fibrosis [8]. Several cases showing overlap phenotypes have been described [5, 6]. The phenotypic variability, including intrafamilial heterogeneity, suggests pleiotropic effects, and variable expression and penetrance.

MEPPC syndrome, caused by *SCN5A* gain-of-function variants, is characterized by frequent atrial and/or ventricular ectopy. In this context, DCM appears to result from a high ventricular ectopy burden [32], as shown in cases with the MEPPC-associated variants R222Q and G213D, where DCM developed only in individuals with a high PVC burden [17, 22, 32]. A systematic review by Peters et al. found that most *SCN5A* variants associated with DCM were also linked to PVCs [10], reinforcing the idea that arrhythmias, whether from gain- or loss-of-function mutations, are central to *SCN5A*-related DCM pathogenesis.

Phenotypic, pathophysiological, genetic, structural, and electrophysiological overlap has been observed between BrS and ARVC [33–35]. This overlap might be due to a shared underlying disease process of the connexome, a protein network essential for mechanical and electrical coupling between cardiomyocytes, integrating several cell-cell junctions and ion channel complexes [36]. NaV1.5, encoded by *SCN5A*, locates to these ion channel complexes [37], linking *SCN5A*-related BrS and ACM to connexome dysfunction. Further studies are needed to determine whether this shared pathophysiology has any impact on patient management [35]. To our knowledge, there are no described cases that presented with both DCM and BrS.

### SCN5A-related mechanisms underlying structural abnormalities

The overlap between arrhythmias and cardiomyopathy raises the question whether the DCM phenotype arises mainly from arrhythmia-induced remodeling or whether it can also result directly from NaV1.5 dysfunction. Studies investigating the consequences of NaV1.5 dysfunction identified several mechanisms that may contribute to cardiac structural remodeling.

Pathophysiological, gain-of-function variants increase intracellular sodium and calcium via the sodium-calcium exchanger, activating pro-fibrotic and hypertrophic signaling pathways such as CAMKII and calcineurin [38, 39]. Some variants induce proton leak, promoting ionic imbalances that not only contribute to arrhythmogenesis, but may also impair excitation-contraction coupling and myofilament function (Fig. 1) [40]. Loss-of-function variants have been associated with elevated TGFβ signaling and myocardial fibrosis in SCN5a-deficient mice, supporting a role for sodium current reduction in TGFβ-mediated structural remodeling through enhanced fibroblast activation, extracellular matrix deposition, and myocardial scarring [41, 42]. NaV1.5 is part of the connexome, where it interacts with, amongst others, cytoskeletal-, adhesion- and desmosomal proteins. It is hypothesized that NaV1.5 dysfunction may impair function or localization of these proteins, compromising myocardial integrity [4]. This also points towards a mechanistic overlap between sodium channelopathies and desmosomal cardiomyopathies [9, 36]. NaV1.5 loss in HL1 cells has been shown to reduce intracellular adhesion strength [43], but the effects of *SCN5A* variants on these interacting proteins remain underexplored. Another hypothesized mechanism involves ectopic expression of the zinc finger protein Snail in transgenic mice, leading to sodium channel downregulation and resulting in a progressive DCM phenotype with conduction disturbances. This model supports a potential role of Snail-mediated ***SCN5A*** repression in the pathogenesis of DCM [44, 45].

Overall, while arrhythmias likely play a significant role in the development of DCM, in vitro studies also support a direct, structural role for *SCN5A* variants in the pathogenesis of cardiomyopathy.

### Recommendations on Genetic Testing of Relatives

The latest European Society of Cardiology (ESC) guidelines recommend genetic testing for first-degree relatives of genotype-positive cardiomyopathy patients to guide follow-up strategies and to exclude non-carriers from unnecessary surveillance [7]. However, specific recommendations for the management of *SCN5A*-related cardiomyopathies are currently lacking, both in affected individuals and asymptomatic carriers.

In pediatric settings, cascade genetic screening has led to the identification of an increasing number of *SCN5A*-positive children ranging from phenotype-negative carriers to those who present with or develop a phenotype [6]. Genetic testing for first-degree relatives of individuals with DCM or ACM is Generally recommended from the age of 10–12 years. Few cases of earlier childhood-onset have been reported [6, 10], where earlier testing may be considered in families with a known history or early-onset disease through shared decision making. However, current evidence on the penetrance of *SCN5A*-related DCM in children under 10 is limited and insufficient to support earlier routine testing at a younger age. An overview of recommendations for clinical management of *SCN5A*-related DCM and asymptomatic *SCN5A* variant carriers can be found in Table 2.

**Table 2:** Recommendations for clinical management of *SCN5A*-related DCM and asymptomatic *SCN5A* variant carriers

	Recommendations	Details	G+/*P*+ (Affected)	G+/*P*- (Asymptomatic Carriers)
Cascade genetic screening	Full pedigree and cascade genetic testing	Allows extension of family-based screening and identification of relatives at risk	- Recommended for all first-degree relatives of *SCN5A* probands, starting at 10–12 years of age - Identifies at-risk carriers, informs surveillance strategy, and allows discharge from clinical follow-up if genotype-negative	
First line cardiac evaluation	- Clinical assessment - 12-lead ECG - Echocardiography - 24-hour Holter Monitoring - Blood tests	Focus on left ventricular dilatation and dysfunction, conduction disorders, arrhythmias, and PVCs	Recommended for diagnosis at the first visit and for monitoring during follow-up (every 1–2 years, or sooner if symptoms or cardiac abnormalities)	Recommended for diagnosis at the first visit and for early detection of abnormalities during follow-up (every 1–3 years before 60 years old, and then every 3–5 years).
Second line diagnostic approaches	Cardiac magnetic resonance imagining	For tissue characterization (although often no LGE, particularly in electrical phenotypes)	Recommended at initial evaluation and for reassessment every 2–5 years	May be considered, especially in the presence of initial structural and/or electrical alterations
Antiarrhythmic Therapy	Based on phenotype and arrhythmic burden	MEPPC: ectopic suppression improves LVEF	- Mexiletine: may be the safest first-line option for PVCs and VT - Class IC AADs (flecainide): highly effective for PVCs, prior ajmaline test for exclusion of BrS may be considered	Not indicated unless phenotype develops
Heart failure related therapy	Based on LVEF and congestion	- SGLT2i ± ACEi/ARB/ARNI, BBs, MRA. - Diuretics if congested	Recommended in case of heart failure (or LV dysfunction)	Not indicated unless phenotype develops
Device Therapy	ICD/pacing therapy based on conduction disease, ventricular arrhythmias and LVEF		Recommended if: - High-grade atrioventricular block - Symptomatic sinus node dysfunction - VT/VF	Not indicated unless phenotype develops
Lifestyle & Restrictions	Activity and drug precautions related to BrS		Avoid Brugada-trigger drugs and manage fever appropriately in cases of overlapping BrS phenotype or *SCN5A*-DCM with BrS family history	No restrictions unless DCM or BrS phenotype develops
Psychological support	Psychological support and genetic counseling in all patients and families affected by inherited cardiomyopathy		Psychological support for coping with diagnosis, potential device implantation, and reproductive implications	Support may be helpful, especially in younger individuals or those with anxiety related to genetic risk or family history

All recommendations regarding cardiac evaluation and genetic screening are based on the European Society of Cardiology guidelines. Lifestyle advice and restrictions related to BrS are based on expertise opinion, as current literature and formal guidelines on these aspects remain limited. Abbreviations: *AAD* antiarrhythmic drugs,* ACEi* angiotensin-converting enzyme Inhibitor, *ARB* angiotensin II receptor blocker, *ARNI* angiotensin receptor–neprilysin inhibitors,* BB* beta blockers, *BrS *brugada syndrome, G*en* genotype, *DCM* dilated cardiomyopathy, *G +* genotype positive (defined as carriers of pathogenic or likely pathogenic variants in *SCN5A)*, *ICD* implantable cardioverter defibrillator, *LGE* late gadolinium enhancement, *LVEF* left ventricle ejection fraction,* MEPPC* multifocal ectopic premature Purkinje-related contractions, *MRA* mineralcorticoid receptor antagonist, *phen* phenotype, *P+/-* phenotype positive/negative, *PVC* premature ventricular contraction,* SGLT2i* sodium-glucose co-transporter 2 inhibitors, *VF* ventricular fibrillation, *VT* ventricular tachycardia

### Diagnostic Assessment and follow-up in genotype-positive, Asymptomatic Relatives

General recommendations for the evaluation of at-risk relatives include cascade Genetic screening with comprehensive pedigree analysis. In case of carriership, cardiac screening consisting of ECG, echocardiography, 24-hour Holter monitoring, and laboratory tests is done [7] (Table 2). Cardiac screening is recommended for diagnosis at the first visit and for early detection of abnormalities during follow-up (every 1–3 years before 60 years old, and then every 3–5 years) [7]. The phenotyping remains fundamental in assessing individuals at risk of a *SCN5A*-related cardiomyopathy. CMR imaging may be considered in genotype-positives [7]. However, *SCN5A*-related DCM rarely demonstrates LGE [10, 29],.

In case of abnormalities, follow-up cardiac evaluation is recommended every 1–2 years, or earlier in the presence of persistent symptoms or documented arrhythmias [7].

In this context, CMR is currently recommended as part of the initial evaluation and for re-assessment every 2–5 years [7].

Given the broad phenotypic spectrum associated with *SCN5A* variants, a comprehensive diagnostic approach is essential. Although knowledge of a variant’s functional impact (gain vs. loss of function) can aid in anticipating associated phenotypes, such information is often unavailable in clinical practice. Phenotype of index patient and family history can be of guidance for clinical evaluation. However, the potential intrafamilial variability in *SCN5A* variant expression underscores the need to consider the full spectrum of *SCN5A*-related phenotypes. When a *SCN5A* variant is identified in a patient presenting with a cardiomyopathy, thorough assessment for conduction disease and arrhythmia, including ECGs and serial Holter monitoring, is warranted. Conversely, in families with a history of primary arrhythmias or conduction disease, longitudinal evaluation should also account for the potential development of a structural cardiomyopathy.

Assessment for BrS using high precordial-lead ECGs may be considered, especially in cases with suspected NaV1.5 loss-of-function. However, given the lack of a clear association between BrS and DCM, pharmacological provocation testing with ajmaline is not routinely recommended. It should be reserved for selected cases with a family history or high clinical suspicion, particularly when initiation of class IC AAD is being considered.

In carriers of *SCN5A* variants, lifestyle advice and clinical management should be individualized. Generally, genotype-positive, phenotype-negative relatives do not require specific BrS-related precautions, such as fever management and drug restrictions, unless there is a documented BrS family history or clinical phenotype consistent with BrS.

### Therapeutic considerations in SCN5A-related cardiomyopathy: a genotype-guided approach

The management of *SCN5A*-related cardiomyopathy requires a genotype-informed approach, as phenotype-based strategies alone might fall short in addressing the arrhythmogenic substrate that plays an important role in disease development and progression in many carriers.

*SCN5A*-related DCM can exhibit remarkable reversibility, particularly in MEPPC syndrome, where Purkinje-origin ectopy contributes to left ventricular dysfunction. Suppression of the arrhythmic burden with class I AAD can lead to normalization of cardiac function (Table 2) [46].

Mexiletine is likely the safest first-line therapy to prevent ventricular arrhythmias and reduce arrhythmic burden in *SCN5A*-related cardiomyopathy. Notably, in overlapping syndromes, mexiletine does not induce ST-segment elevation, whereas flecainide has been reported to do so [47, 48]. An exceptional response to class IC AAD, especially flecainide, has been observed in reducing the burden of premature ventricular complexes [10, 46]. Li et al. demonstrated that in MEPPC syndrome, upregulation of β1 and β3 subunits increases sodium channel excitability in Purkinje fibers, thereby elevating arrhythmia susceptibility but also enhancing sensitivity to class IC AAD [49]. Although uncommon, mixed phenotypes, such as latent conduction system disease or BrS, can coexist with DCM [5]. Therefore, ambulatory *SCN5A* DCM patients considered for class IC AAD should first undergo sodium channel blocker challenge testing with ajmaline preferably in a center of expertise with BrS [47]. Given the complexity of managing such mixed phenotypes, functional studies are essential to characterize variant effects and guide treatment.

In *SCN5A*-related DCM caused by loss-of-function variants, pacemaker implantation or pacing-ICD is indicated for patients with high-grade atrioventricular block or symptomatic sinus node dysfunction [50]. For patients with DCM and LQT, treatment should include mexiletine, eventually combined with nadolol, and consideration of ICD implantation [47].

Psychological support and genetic counseling are recommended for all families affected by inherited cardiomyopathy. This provides tailored assistance with coping with the diagnosis, device-related decisions, family planning, and supportive care as needed, particularly for the young or anxious (Table 2).

### Future Directions

It should be noted that the applicability of current ESC guidelines recommendations to *SCN5A*-related cardiomyopathies may not always fit appropriately, given the lack of disease-specific evidence. Large-scale, longitudinal studies are needed to better delineate the phenotypic spectrum and clinical course, and to refine evidence-based recommendations for *SCN5A*-related cardiomyopathy. Observational studies including genotype-positive, phenotype-negative individuals may offer valuable insight into disease penetrance, progression, and potential genetic or environmental modifiers. Crucially, future research should clarify the underlying mechanisms driving *SCN5A*-associated DCM, specifically whether it arises mainly from intrinsic myocardial structural abnormalities or electrical instability. Understanding the biophysical properties of specific *SCN5A* variants is essential for personalized monitoring, therapeutic decisions, and the development of targeted interventions.

Risk prediction tools for arrhythmic events in *SCN5A*-related cardiomyopathy are currently lacking. Developing such models is essential to guide decisions on protective therapies, including ICD placement.

From a reproductive standpoint, preimplantation genetic testing (PGT) is available for high-risk variants in genes such as *PLN*, *LMNA*, and *FLNC*tv [51, 52]. For genes like *SCN5A*, limited data on penetrance and risk prediction complicates estimation of risk reduction by PGT. These cases are therefore typically assessed individually by multidisciplinary teams, considering disease expression, severity, and penetrance of individual families. However, as evidence grows and genotype-phenotype correlations strengthen, *SCN5A* may also emerge as a more actionable gene in reproductive counseling and PGT decision-making.

## Conclusions

*SCN5A*-related cardiomyopathies encompass a broad clinical spectrum and are often preceded or accompanied by conduction disease, as well as atrial and ventricular arrhythmias, such as PVCs in the context of MEPPC. In rare cases, overlap syndromes involving DCM and *SCN5A*-related cardiac channelopathies such as LQT3 and PCCD occur. The fact that electrical abnormalities often arise before the onset of *SCN5A*-related DCM highlights the role of arrhythmias in the development of the DCM phenotype.

Cascade genetic testing aids in identifying at-risk individuals, enabling earlier intervention when necessary to reduce morbidity and mortality risk. The clinical expression of *SCN5A* variants is highly variable, with incomplete penetrance and intrafamilial heterogeneity, underscoring the role of modifying genetic and environmental “second hit” factors yet to be fully elucidated. Although family history can guide clinical evaluation, diagnostic assessment should remain comprehensive, addressing the entire spectrum of *SCN5A*-related phenotypes. While arrhythmia management is a key therapeutic focus in *SCN5A*-related DCM with a high burden of ventricular ectopy, cases of DCM without overt arrhythmia or conduction disease have been described. Emerging insights into the biophysical properties of the NaV1.5 sodium channel and its pathogenic mechanisms suggest that *SCN5A* variants also directly contribute to myocardial structural damage, an area warranting further investigation.

Future research should prioritize a deeper understanding of the *SCN5A*-related cardiomyopathy phenotype, the development of risk stratification tools for arrhythmic events, and the elucidation of underlying disease mechanisms. Building on these insights, efforts may focus on exploring targeted therapies to improve prognosis and patient care. Together, these efforts will advance precision medicine in *SCN5A*-related cardiomyopathies.

Abbreviations: BrS = Brugada Syndrome; CA/SCD = cardiac arrest/sudden cardiac death; DCM = dilated cardiomyopathy; LVEF = left ventricular ejection fraction; PCCD = progressive cardiac conduction disease; PVCs = premature ventricular complexes; SSS = sick sinus syndrome.

## Key References

- Peters S, Thompson BA, Perrin M, James P, Zentner D, Kalman JM, et al. Arrhythmic Phenotypes Are a Defining Feature of Dilated Cardiomyopathy-Associated SCN5A Variants: A Systematic Review. Circ Genom Precis Med. 2022;15(1):e003432. doi: 10.1161/circgen.121.003432.
  - This systematic review examines clinical features of SCN5A-related DCM, linking variants to different phenotypes. It also outlines key therapeutic approaches for associated arrhythmias.
- Ahamed H, Gopi A. Effect of Flecainide on Multifocal Ectopic Purkinje-Related Premature Contractions in an R814W SCN5A Carrier. JACC Case Rep. 2024;29(5):102223. doi: 10.1016/j.jaccas.2024.102223.
  - This emblematic case describes a gain-of-function SCN5A variant linked to a high burden of ventricular ectopy and DCM, with a remarkable response to class 1 C antiarrhythmic therapy leading to full recovery of left ventricular function.
- Hermida A, Jedraszak G, Ader F, Denjoy I, Fressart V, Maury P, et al. Systematic analysis of SCN5A variants associated with inherited cardiac diseases. Heart rhythm. 2025;22(3):844 − 51. doi: 10.1016/j.hrthm.2024.08.018.
  - The authors report several families exhibiting overlapping phenotypes of DCM with cardiac conduction disorders, Brugada syndrome and other cardiac disorders. The findings highlight the concepts of incomplete penetrance and interfamilial heterogeneity.

## Data Availability

No datasets were generated or analysed during the current study.
